# Porous Scaffold Design for Additive Manufacturing in Orthopedics: A Review

**DOI:** 10.3389/fbioe.2020.00609

**Published:** 2020-06-17

**Authors:** Hao Chen, Qing Han, Chenyu Wang, Yang Liu, Bingpeng Chen, Jincheng Wang

**Affiliations:** ^1^Department of Orthopedics, Second Hospital of Jilin University, Changchun, China; ^2^Department of Dermatology, The First Hospital of Jilin University, Changchun, China

**Keywords:** additive manufacturing, orthopedic scaffolds, porous structure design, cellular design, mechanical property

## Abstract

With the increasing application of orthopedic scaffolds, a dramatically increasing number of requirements for scaffolds are precise. The porous structure has been a fundamental design in the bone tissue engineering or orthopedic clinics because of its low Young’s modulus, high compressive strength, and abundant cell accommodation space. The porous structure manufactured by additive manufacturing (AM) technology has controllable pore size, pore shape, and porosity. The single unit can be designed and arrayed with AM, which brings controllable pore characteristics and mechanical properties. This paper presents the current status of porous designs in AM technology. The porous structures are stated from the cellular structure and the whole structure. In the aspect of the cellular structure, non-parametric design and parametric design are discussed here according to whether the algorithm generates the structure or not. The non-parametric design comprises the diamond, the body-centered cubic, and the polyhedral structure, etc. The Voronoi, the Triply Periodic Minimal Surface, and other parametric designs are mainly discussed in parametric design. In the discussion of cellular structures, we emphasize the design, and the resulting biomechanical and biological effects caused by designs. In the aspect of the whole structure, the recent experimental researches are reviewed on uniform design, layered gradient design, and layered gradient design based on topological optimization, etc. These parts are summarized because of the development of technology and the demand for mechanics or bone growth. Finally, the challenges faced by the porous designs and prospects of porous structure in orthopedics are proposed in this paper.

## Introduction

The bone structure consists of cortical and cancellous bone. Bone is a non-uniform porous structure, the density of which gradually increases from the inner cancellous bone to the outer cortical bone. At present, the porous designs of orthopedics are trying to imitate the structure of bone from the following aspects, such as Young’s modulus, compression strength, biocompatibility, and bone ingrowth (Weiner et al., 1999; Gómez et al., 2016; Fantini and Curto, 2017).

Among which, bone ingrowth and mechanical properties are essential parts of orthopedic scaffolds (Nune et al., 2017b; Ran et al., 2018). Effective bone ingrowth can significantly reduce the rate of revision (Warnke et al., 2009). The main factors affecting bone ingrowth are the following: porosity, pore size, the shape of the pore, and randomness of pore distribution. The porous structure with suitable pore size and porosity brings enough space for cell proliferation (Kuboki et al., 2001; Zadpoor, 2015). Different pore shapes can lead to altering permeability, which results in different bone ingrowth (Arabnejad et al., 2016; Dallago et al., 2018; Arjunan et al., 2020). The randomly distributed pore is similar to the internal structure of the bone (Liang et al., 2019). Although some newly developing randomized structures, such as Voronoi, can imitate the structure of bone well, the discussion on the structure of randomization is seldom. Early stress stimulation plays a crucial role in long-term bone healing response (Klosterhoff et al., 2020). The stress shielding effect can be effectively reduced because the scaffolds have a similar Young’s modulus to the bone. Many types of researches showed that a negative correlation occurs between Young’s modulus and compressive strength (Zadpoor, 2015). The relationship between them can be balanced by adjustment of porosity and pore shape (Torres-Sanchez et al., 2017; Zhao et al., 2017). In the meantime, various pore shapes lead to different failure directions under ultimate stress. Thus, orthopedic scaffolds are required to appropriate pore size and porosity, reasonable pore shape, and random or gradient pore distribution analogous to bone (Hao et al., 2016; Nune et al., 2017c; Wu et al., 2017).

In order to achieve the above objectives, most researchers use traditional methods such as space-holder method, fiber sintering method, and freeze casting method (Fang, 2016). These manufacturing methods have the advantages of small pore, easy to manufacture, and suitable for large-scale manufacturing. However, these methods are not controllable in both macro shape and micropore (Han et al., 2019). With the development of AM, the pore shape, pore size, porosity, and the macro shape can be controlled by computer design in advance. In AM technology, three-dimensional (3D) objects are created, adding materials layer by layer with computer-aided design (CAD) (Zopf et al., 2015; Hao et al., 2016). In this process, some geometry-based structures at the level of cellular design, such as diamond/face-centered-cubic (FCC), body-centered cubic (BCC)/octahedron (OC) and rhombic dodecahedron (RD), etc., can be precisely manufactured. In addition to these structures, the parametric design can only be achieved through AM technology, such as Triply Periodic Minimal Surface (TPMS) and Voronoi. These algorithm-based structures have some advantages such as high randomization, functional internal connectivity, and excellent mechanical properties. Nevertheless, these structures have not been adequately studied. The whole shape can also be achieved to controllable gradients in AM technology. The gradient scaffold is the same as the whole structure of natural bone, which shows excellent mechanical property distribution and bone growth performance. Gradients have different forms. Topology optimization (TO) is a mathematical method that optimizes material layout within a given design space, which is a vital part of the design for AM. In order to obtain the mechanics more reasonable distribution, the design of the whole structure is divided into different density layers through topology optimization.

It is meaningful to discuss all the above structures. There were many reviews on the porous structure of orthopedics previously. Some reviews focused on cellular structure design (Atesok et al., 2016; Savio et al., 2018). A summary of some relevant ideas in various cellular designs and AM of the cellular structure was presented in 2019 by Nazir et al. (2019). Savio et al. (2018) discussed the existing cell structure by the geometric point of view. Pałka and Pokrowiecki (2018) described the design and optimization process of the porous structure. Wang et al. (2016) detailed discussed the topological design of various porous metals. In their review, various unit cells that appeared before 2016 are described, as well as the mechanical and biological performance of titanium alloy and other metals (Wang et al., 2016). The mechanical differences between unit cells were needed to be understood. Other reviews focused on the whole design of the porous structure. Babaie and Bhadur (2017) discussed the effect of porosity on biology and mechanical properties from a macroscopic point of view. Zhao et al. (2017) studied the main mechanical properties of porous structure and the effect of porosity on mechanical properties in detail. However, few researchers have addressed the problem in a systematic and regular summary of the design of porous structures and the mechanical and biological changes with the design. In our review, porous structures were divided into cellular design and the whole design. The cellular design was divided into parametric design and non-parametric design according to whether the structure is constituted by algorithm or not. As the porosity changes of scaffold, the whole design of scaffold was divided into uniform design, gradient design, and TO based design. Different designs and the changes of mechanical and biological properties caused by these designs were mainly reviewed.

Here in this review, porous scaffold designs and porous designs in other fields may apply in orthopedic were discussed.

## Cellular Design

The unit cell is the basis of the porous structure at a microscopic point using AM technology. Designs are divided into non-parametric design and parametric design. Non-parametric design is structural and geometric design. Parametric design is that cellular structures which are generated according to specific algorithms. It is worthy of discussing the performance differences between various designs.

### Non-parametric Design

Non-parametric design can also be called based geometry design. According to the different shapes, the non-parametric design was divided into the 3D structural based design and the plane structural based design. The Diamond/FCC, the BCC, and the other polyhedron structure were parts of the design based on the 3D structure. Honeycomb was the most common plane structural based design. These structures were discussed in terms of design, mechanical, and biological properties.

#### The BCC/OC

**FIGURE 1:**
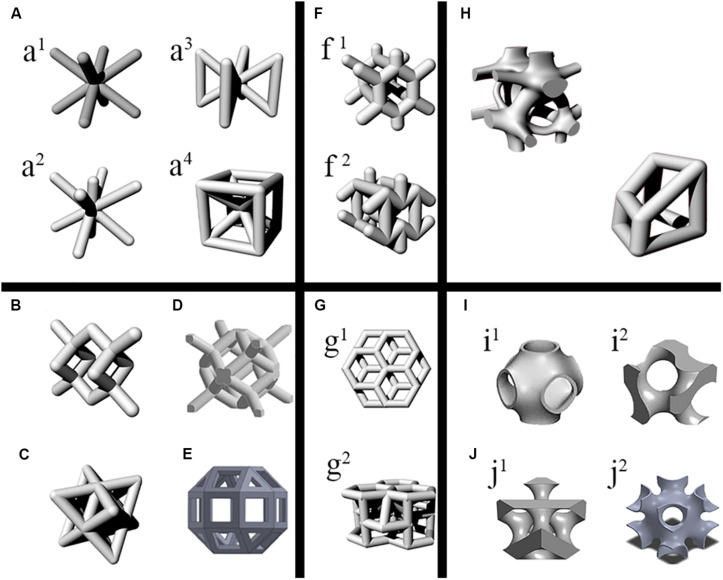
Units of various non-parametric and parametric designs. **(A)** The body-centered cubic (BCC) unit and its modified units. **a^1^**: BCC unit. **(a^2^)** A type of modified BCC unit by Adding vertical stiffeners through the center of the BCC/OC unit. **(a^3^)** The BCCZ unit. **(a^4^)** The pillar BCC. **(B)** The Diamond unit. **(C)** The Octet unit. **(D)** The rhombic dodecahedron unit. **(E)** The rhombic cube octahedron unit. **(F)** The Honeycomb units. **(f^1^)** The commonly Honeycomb unit. **(f^2^)** The modified Honeycomb unit. **(G)** A new type Honeycomb unit. **(g^1^)** Positional relationship of adjacent layers of new type Honeycomb unit. **(g^2^)** Structural characteristics of the layered slice and rod-connected structure of new type Honeycomb unit. **(H)** The Voronoi units. **(I,J)** The classification of TPMS**. (I)** The surface dominated by stretching. **(i^1^)** Primitive surface. **(i^2^)** I-WP surface. **(J)** The surface dominated by bending. **(j^1^)** Diamond surface. **(j^2^)** Gyroid surface.

Although the BCC/OC is a relatively simple design, it has two advantages that make it a frequently used type. First, the BCC/OC structure could be easy to design. Second, the BCC/OC structure could be well-manufactured because of inclining all struts properly for minimizing the warping effect during the SLM process. The original BCC/OC is obtained by connecting the center of hexahedron with eight vertices (shown in Figure 1a^1^).

Through BCC/OC design, the scaffold could significantly reduce Young’s modulus. The mechanical tests of scaffolds designed with BCC/OC and solid structures were carried out at the same time. The results showed that Young’s modulus of the orthopedic scaffold could be reduced by 75–80% through BCC/OC design (Wang L. et al., 2017). Moreover, the scaffolds designed by BCC/OC design had highly predictable size effects, which might be related to the high accuracy of manufacturing BCC structure. Due to the predictability of size effect, the mechanical properties corresponding to porosity can be accurately inferred. Consequently, BCC/OC designed scaffold was usually in accordance with the expected mechanical properties (shown in Table 1) (Yang, 2015). The strut cross has existed in the BCC/OC unit cell. When the fluid passed through the cross, the speed was slowed down. Generally, the low flow rate was conducive to cell proliferation. Thus, the BCC/OC designed scaffold had advantages in bone ingrowth (Limmahakhun et al., 2017a). However, the compressive properties of the BCC/OC designed scaffold were not satisfactory.

**TABLE 1:** The mechanical properties of various cellular designs.

Design		Materials	Porosity (%)	Mechanical properties			References
				Young’s modulus (GPa)	Yield stress (MPa)	Compressive strength (MPa)	
Cortical bone		–		14.0 ± 9	109.60 ± 4.70	202 ± 38	Gu and Zheng (2010)
Cancellous bone		–		0.79 ± 0.78	55.30 ± 8.60	–	Gu and Zheng (2010), Bobbert et al. (2017)
				4.59 ± 1.60	–	–	Choi et al. (1990)
Body centered cubic			33.78 ± 0.01	9.0 ± 0.6	392 ± 14	532 ± 11	
		Ti6Al4V	53.06 ± 0.01	4.6 ± 0.4	192 ± 14	256 ± 4	
			71.87 ± 0.01	1.6 ± 0.2	53 ± 4	74 ± 2	
			51.90 ± 0.02	3.5 ± 0.5	86 ± 11	128 ± 8	Onal et al. (2018)
		Polyamide	Graded 0.74–0.89	0.01	0.01	–	Maskery et al. (2016)
BCCZ		Polyamide	Graded 0.74–0.89	0.37	0.01	–	
Face Centered Cubic/Diamond			–	4.24	99.64	–	
			64	3.70	70.60 ± 7.00	113.00 ± 17.30	Ahmadi et al. (2014, 2015), Amin Yavari et al. (2015), Kadkhodapour et al. (2015)
		Ti6Al4V	72	2.20	31.70 ± 13.00	57.00 ± 12.80	
			79	1.50	28.90 ± 6.20	46. 50 ± 2.50	
			89	0.50	6.80 ± 2.30	15.10 ± 0.30	
			33.8 ± 0.8	3.70 ± 0.20	–	115.20 ± 12.80	Li et al. (2016)
		Ti6Al4V	50.9 ± 0.6	2.30 ± 0.10	–	51.50 ± 6.40	
			61.3 ± 0.4	1.70 ± 0.20	–	33.10 ± 5.40	
		Iron	77.7 ± 1	1.70 ± 0.10	22.50	–	Li et al. (2018b)
		Magnesium	64 ± 0.2	0.70 ± 0.10	15.00	–	Li et al. (2018c)
		Ti6Al4V	Graded 21.0 to 91.3	19.60–0.60	227.90–17.90	295.90–22.10	Zhang et al. (2019)
FCCZ		Ti35Zr28Nb	83.2 ± 2.3	1.1 ± 0.1	–	27 ± 2	Li Y. et al. (2019)
FBCCZ		Ti35Zr28Nb	49.9 ± 3.2	1.3 ± 0.1	–	58 ± 3	
The Rhombic Dodecahedron		Ti6Al4V	75	0.95 ± 0.05	–	50.00 ± 0.90	Liu et al. (2016)
		Ti24Nb4Zr8Sn	70	4.36	–	–	Nune et al. (2017b)
		Ti6Al4V	62.1	6.30 ± 0.10	–	112.00 ± 2.80	Zhao et al. (2016)
TPMS	P surface	Photopolymer resin	30	0.15	3.10	–	Afshar et al. (2016, 2018)
			60	0.49	26.10	–	
		Ti6Al4V	62	11	–	–	Du Plessis et al. (2018)
		Photopolymer resin	Graded 30–60	0.35	8.00	–	Afshar et al. (2016, 2018)
	D surface	Ti6Al4V	5	17.19	1581		Yuan et al. (2019)
			10	15.73	1342		
		Photopolymer resin	30	336.00	14.60	–	
			60	79.50	3.50	–	
		Ti6Al4V	80	1.25 ± 0.07	69.21 ± 4.22	–	Yuan et al. (2019)
			95	0.12 ± 0.03	4.66 ± 0.13	–	
		Photopolymer resin	Graded 30 to 60	170.00	3.60	–	Afshar et al. (2016, 2018)
	G surface	Ti6Al4V	5	19.14	1581		Yuan et al. (2019)
			10	17.45	1342		
		Ti6Al4V	70.99 ± 9.3	10.60 ± 0.28	22.44 ± 0.46	–	Zaharin et al. (2018)
			77.86 ± 8.2	5.61 ± 0.36	11.25 ± 0.31	–	
		Ti6Al4V	85	1.13 ± 0.53	41.0 ± 3.9	–	Yang et al. (2018)
			95	0.13 ± 0.04	5.2 ± 0.5		
	I-WP surface	Photopolymer resin	Graded 40 to 60	234.8	10.00	–	Afshar et al. (2016, 2018)
The Voronoi		Ti6Al4V	70	3.92	–	158	Liang et al. (2019)
			Graded 60 to 95	0.14–2.37	–	1.94–116.61	Wang et al. (2018)
			Graded 50 to 85	2.13–3.97		78.9–130.5	Du et al. (2020)

**FIGURE 2:**
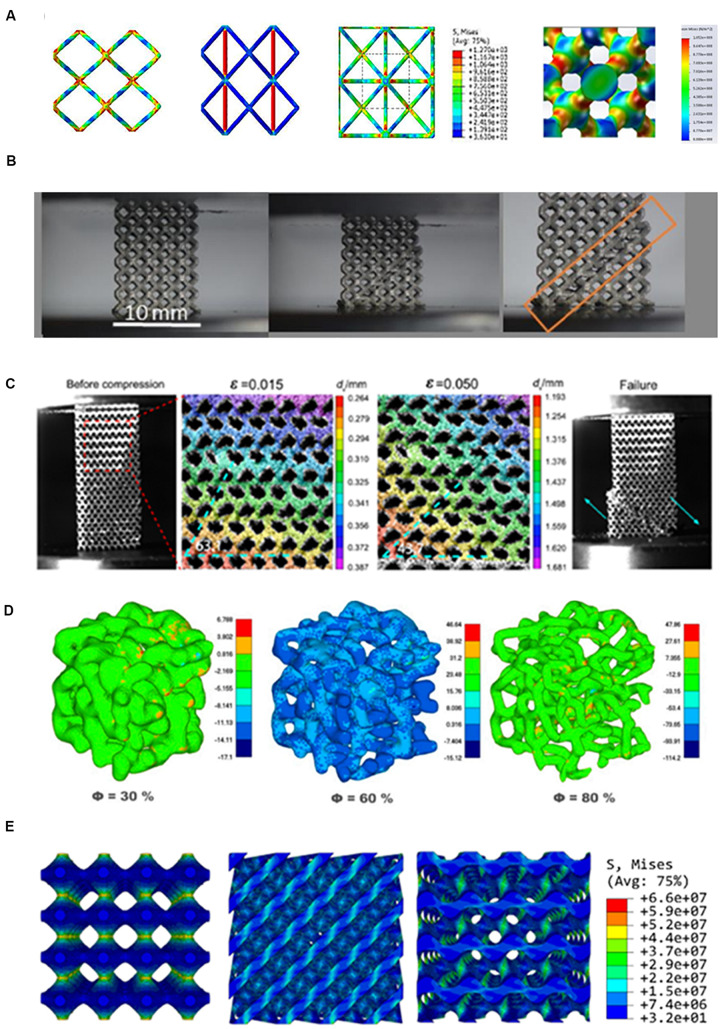
Stress distribution and failure mechanism analysis of non-parametric design and parametric designs. **(A)** From left to right, the stress distribution of body-centered cubic (BCC), modified BCC unit, pillar BCC and diamond units. The red part represents the stress concentration area. Failure modes of **(B)** BCC, **(C)** Diamond. The stress distribution of **(D)** Voronoi, **(E)** TPMS. Reprinted: **(A)** from Zhang B. et al. (2018) with permission from Elsevier; **(B)** from Onal et al. (2018) under the terms of the Creative Commons Attribution License; **(C)** from Zhang et al. (2019) with permission from Elsevier; **(D)** from Sharma et al. (2019) under the terms of the Creative Commons Attribution License; **(E)** from Maskery et al. (2018b) under the terms of the Creative Commons Attribution License.

Maskery et al. (2016) proposed a BCC/OC based reinforced scaffold with four reinforcements in the *z*-axis direction, which named BCCz (shown in Figure 1a^3^) (Beyer and Figueroa, 2016). Similarly, the eight vertices of the BCC/OC unit were connected to obtain the reinforced structure, which is called pillar BCC (shown in Figure 1a^4^). It combined the characteristics of the cube and BCC/OC (Limmahakhun et al., 2017b; Wang L. et al., 2017). Adding vertical stiffeners through the center of the BCC/OC unit was also a way to improve the original BCC/OC (shown in Figure 1a^2^) (Feng et al., 2016). All these reinforced BCC/OC scaffolds were better than the original BCC structures in compressive properties and Young’s modulus. It should be noted that the addition of stiffeners may change the anisotropy and fatigue life. The combination of FCC and BCC, named face and body-centered cube cell (FBCC)/with vertical struts (FBCCz), was another way of improvement. The mechanical properties were measured in the compression test, and the results indicated that FBCC/FBCCZ designed scaffold has higher stiffness than the original BCC/OC (Feng et al., 2016). Furthermore, the FBCCz designed scaffold showed the highest specific modulus, which meant that the structure provided superior performance for compressive load scenarios that attempt to optimize the stiffness-to-weight ratio (Mazur et al., 2015). The research on the stress concentration point of BCC/OC was significant for the subsequent optimization. The stress was mainly concentrated at the cross of the struts, which is found in the result of the FEA (shown in Figure 2). A spherical-node-body-centered-cubic unit was developed to reduce1 the stress concentration and improve the stiffness. The structure was based on the BCC/OC stress concentration point, which was changed the joint from the original cube structure to the filet structure. The structure can not only significantly improve the stiffness of the porous structure but also reduce the stress concentration of joints (Ren et al., 2019). The mechanical properties of the BCCz and FBCCz were shown in Table 1. Most of the previous studies focused on mechanical properties but paid relatively little attention to biological properties. This might be related to the fact that porosity is the main factor affecting bone ingrowth. The pillar OC performed better than the OC in the aspect of bone ingrowth. The pillar OC had a larger relative surface area. Cell proliferation test was conducted in pillar OC and OC. As expected, the rate of pre-osteoblastic cell proliferation was revealed that the pillar OC had significantly effective compared with the OC. There were few literatures on bone ingrowth of other reinforced structures.

As the orthopedic scaffold designs, BCC and its reinforced design had the advantages of the excellent mechanical properties and easy to manufacture characteristics. It also had some disadvantages, such as insufficient internal surface area and relatively low anisotropy. In general, BCC and its reinforced designed scaffold were a kind of choice that was easy to design and manufacture (Limmahakhun et al., 2017a).

#### The Diamond/FCC

The diamond cell was one of the typical unit cells for AM in Orthopedics. The Diamond cell possessed FCC elemental configuration possessing tetrahedral angles of 109° between elements (shown in Figure 1B). The Diamond/FCC cell had fourteen vertices and sixteen equal edges. The length of a struct (L), length of the unit cell (∝), and the angle between struts and the horizontal plane (θ) were related to the formula as follows (Ahmadi et al., 2014).

α=22Lcos θ θ=35.26 α=43L3

Its stability can be seen from the structural design. Young’s modulus, compressive strength, deformation mechanism, and fatigue life were used to verify the applicability of the Diamond/FCC as orthopedic scaffolds. From the following aspects, it can be confirmed that the scaffold designed with Diamond/FCC had similar Young’s modulus to natural bone. Ahmadi et al. (2014) and Neff et al. (2015) evaluated the mechanical property of the Diamond/FCC structure built by Ti6Al4V on analytical solutions and finite element analysis (FEA). The results of the two methods showed that Young’s modulus could be effectively reduced by Diamond/FCC design (Ahmadi et al., 2014; Neff et al., 2015). In the compression test, the mechanical properties of porous Ti6Al4V scaffolds in Diamond/FCC structure were evaluated, and similar conclusions were obtained that Young’s modulus was similar to natural bone (Li et al., 2016; Yanez et al., 2016). The Diamond/FCC designed scaffold performed well with different porosity. The structure of porous Diamond/FCC with low porosity was similar to cortical bone in Young’s modulus and compressive strength, while these properties in high porosity were similar to cancellous bone (Zhang B. et al., 2018). The Young’s modulus, which matched the height of bone, showed that the mechanical properties of Diamond/FCC designed scaffolds were suitable for bone implantation. In the degradable materials, the Diamond/FCC designed scaffold also showed excellent mechanical properties. Now magnesium (Mg) and iron (Fe) were mostly used in degradable materials, while the Mg/Fe scaffolds designed by Diamond/FCC still had excellent mechanical properties after a period of degradation. Li et al. (2018b) explored the mechanical properties of degradable porous Mg designed with Diamond/FCC after biodegradation *in vitro*. The Young’s modulus (700–800 MPa) of the porous scaffolds was found to fall into the range of the cancellous bone even after four weeks of biodegradation in the results (Li et al., 2018b). Li et al. (2018a) designed porous scaffolds with Diamond/FCC, which used iron with slower degradation, and they evaluated the time-dependent mechanical properties. The results illustrated that Young’s modulus of porous iron was still within the range of cancellous bone after four weeks of biodegradation. The excellent mechanical properties were also performed in degradable scaffolds with Diamond/FCC design (Li et al., 2018a). The mechanical properties of the Diamond/FCC were shown in Table 1. Most researches on orthopedic experiments studied more on the mechanical properties, and less on biological properties, scarcely any research on *in vitro* experiments. Li et al. (2016) evaluated the biocompatibility *in vitro* and osseointegration *in vivo* of porous Ti6Al4V designed in Diamond/FCC with different pore sizes. In their research results, the Diamond/FCC structure with a pore of 300–400 μm was the most suitable for bone integration and biocompatibility (Li et al., 2016). In the research of Wang et al. (2019), the Diamond/FCC designed porous Ti/Ta showed excellent osteogenesis when the pore diameter is 500 μm. In the study of Taniguchi et al. (2016), it was found in a porous Ti designed with Diamond/FCC that the pore diameter of 600 μm was the suitable size both in bone ingrowth and mechanical property. The reason for this difference may be related to the different materials used in manufacturing. At the same time, it also indicated that the scaffold with reasonable pore diameter designed with Diamond/FCC was suitable for the adsorption and proliferation of osteoblasts. Zhang B. et al. (2018) studied the implanting of scaffolds based on Diamond/FCC design *in vivo*. The results showed that new bone tissue grew inwards in cancellous bone and dense bone after implantation of porous Diamond/FCC structure in 4 months (Zhang L. et al., 2018). These results showed that the Diamond/FCC structure was suitable for bone ingrowth.

Because the porous scaffolds in the human body were under cyclic loading conditions during walking and running, the fatigue behavior was an essential part of bone implants. The analysis of the failure mechanism can partly reflect fatigue behavior (Li et al., 2017). To obtain the failure mechanism, the porous scaffolds needed to be tested under fatigue for a long time (Hedayati et al., 2016a). In the case of increasing stress, the failure mechanism of the Diamond/FCC structure was worth studying. In the Diamond/FCC unit cell, the bending of internal struts was expected owing their inclination relative to compress direction. FCC/diamond designed scaffold had a 45° shear band under high stress (shown in Figure 2). It indicated that designed by Diamond/FCC on the scaffolds were isotropic in the mechanical properties (Ahmadi et al., 2015; Kadkhodapour et al., 2015; Zargarian et al., 2016; Maskery et al., 2018a). To explore the cause of this failure mechanism, the FEA was used. In the FEA results of the Diamond/FCC unit, the struts between the unit cell were where the Diamond/FCC stress was enormous (shown in Figure 2). It illustrated that the local state of stress influent fatigue behavior and a useful direction for improving the fatigue performance of porous structure designed with Diamond/FCC (Van Hooreweder et al., 2017; Zhang B. et al., 2018). An optimized Diamond/FCC was designed by optimizing the smooth surface at the nodes based on the optimization of local structure. The results of the compression test showed that the structural strength of the optimized Diamond/FCC was significantly improved (Liu et al., 2018). In different situations, orthopedic scaffolds were needed in various mechanical properties. The optimized Diamond/FCC was designed by eliminating the struts corresponding to two parallel faces for better bending performance (Alana et al., 2019). An optimized FCC with longitudinal struts was designed to obtain higher longitudinal compressive properties. The results illustrated that the longitudinal mechanical properties of scaffolds with 83% porosity were higher than the transversal mechanical properties (Li Y. et al., 2019). Few studies had focused on these optimized structures for bone ingrowth.

The Diamond/FCC design is a kind of cellular design with stable, excellent mechanical properties and good bone growth. Remarkably, the mechanical performance of the scaffold designed by Diamond/FCC was almost the same in different directions. The Diamond/FCC designed scaffolds could be applied to the situation of under multidirectional stress in orthopedics.

#### The Other Polyhedron Structures

In addition to Diamond/FCC and BCC/OC structure, other polyhedral structures commonly used in orthopedics included the RD, truncated cube (TC), Octet, and rhombic cube octahedron (RB).

The RD was a central symmetric structure, which showed the same mechanical properties in the three principal directions (shown in Figure 1D) (Xiao et al., 2015). The compressive strength and Young’s modulus of the RD were closed to the cancellous bone in high porosity (shown in Table 1) (Liu et al., 2016; Nune et al., 2017b). As an orthopedic scaffold, fatigue life was required to be enough. The failure mechanism of the RD structure was observed in the compressive test. The results showed that a shear band appeared along the 45° direction of the scaffold results from bending and damage of support struts (Xiao et al., 2015, 2017; Zargarian et al., 2016). The possible reason for this phenomenon was that the stress was concentrated at the intersection of the struts, and the struts had an inclined angle (Zhao et al., 2016). RD-designed scaffolds had excellent biological properties. RD-designed scaffolds provided the necessary nutrition and oxygen supply for cells and provided an excellent osteogenic microenvironment for the integration of osteoblasts in the results (Nune et al., 2017b).

In recent years, there were few studies on other polyhedron structure. Through the FEA and compression test of Octet structure (shown in Figure 1C), it was found that its mechanical properties were near to that of bone under the appropriate porosity (Bagheri et al., 2018). Porous RB (shown in Figure 1E) made of Ti-24Nb-4Zr-8Sn had been tested *in vitro*. It showed excellent proliferation and differentiation of osteoblasts, and it can form a mineralized bone-like extracellular matrix by secreting bone markers.

These studies indicated that the polygon designs were easily obtained, while its mechanical and biological properties were suitable for orthopedic scaffolds.

#### Honeycomb

The honeycomb structure applied to the aerospace field in the early stage. With the attention of researchers to favorable properties, such as low weight, high stiffness, and high porosity, numerous applications of Honeycomb structure as structural and biomedical materials had been found. The diagram of the honeycomb units is shown in Figure 1F.

The relationship between the mechanical properties and honeycomb structure was studied in FEA. By adjusting the porosity of the honeycomb structure, Young’s modulus can be controlled between cortical bone and cancellous bone (Golodnov et al., 2018; Zhao et al., 2018). By comparing the failure mechanism of the simple cube and honeycomb structure, and it can be seen that the failure of honeycomb structure was different in two directions under two kinds of vertical pressure (Choy et al., 2017). Due to the mechanical differences in different directions, Peng et al. (2019) proposed a new type of honeycomb structure (shown in Figures 1g^1^,g^2^), which uses layered and flaky bars to connect honeycomb. The new honeycomb structure can imitate the mechanical properties of cancellous bone well (Peng et al., 2019).

The honeycomb structure had low weight and high stiffness. However, as the mechanical properties of honeycomb structure vary significantly in different inclinations (directions), the honeycomb structure needs to be improved in the future (Wang Z. et al., 2017; Zhao et al., 2018).

### Parametric Design

With the advancing AM, simple structures are no longer satisfied with the needs of the experiment or clinic. Various complex structures can be made by AM technology (Nazir et al., 2019). According to algorithms is the advanced method to build a porous structure. The current problems can be avoided through different algorithms, such as surface smooth, randomization, strut thickness, and interface mismatch (Bobbert et al., 2017; Tehrani et al., 2018; Montazerian et al., 2019; Yuan et al., 2019). A series of methods based on algorithms is proposed to build a complex porous structure.

There are two main methods to design the porous structure according to algorithms, Voronoi-Tessellation, and TPMS.

#### Based on Voronoi

Many pieces of research showed that optimum porous design of bone scaffolds should copy natural bone properties (Zhang et al., 2016; Fantini and Curto, 2017). The Voronoi structure is similar to the microstructure of bone in morphology. Voronoi generates a mesh structure based on random discrete points. These random points are reasonably connected to be a network structure (Liu et al., 2019). The design of the porous structure based on two-dimension (2D) Voronoi had already been proposed by Kou and Tan in 2010. The Voronoi was linked to orthopedic scaffolds in their study. They created irregular and random scaffolds, which were merged Voronoi cells. The vertices of which were modeled as control points of closed B-Spline curves. The B-spline curves were employed to represent the boundaries of the irregular shaped pores. In their study, it merely showed that the Voronoi structure was closer to the appearance of bone structure. The specific similarity with bone was not reflected in their study (Kou and Tan, 2010). With the profound study on Voronoi, researches had achieved significant progress. A method of reverse creating Voronoi structure was proposed, which was establishing the Voronoi model through irregular points of computed tomography (CT) data extraction. The results inspired a new idea, in which a bone-like Voronoi model was generated according to the average value of statistic irregular points in CT data based on big data (Yang and Zhao, 2017). In addition to building the Voronoi model from CT data, the Voronoi model can be built by other methods directly. The isotropic interconnected porous models were designed with Voronoi tessellation. The leading morphology indices included the trabecular thickness, trabecular separation, trabecular number, bone volume to total volume ratio, and the bone surface to bone volume ratio. These indices were precisely matched to trabecular bone (Gómez et al., 2016; Fantini and Curto, 2017). However, the Voronoi structure built in this method had inevitable problems such as poor repeatability, energy-consuming, and long-time cycle (Wang et al., 2018; Du et al., 2020). A top-down design method based on Voronoi-Tessellation was proposed, which controlled the irregularity of the Voronoi designed porous structures by a random coefficient. The controllable Voronoi structure means controllable pore diameter, porosity, and struct width to imitate bone. So, the Voronoi designed scaffold was conformed to the cancellous bone in appearance. The Voronoi designed unit cells were shown in Figure 1H.

In the bionics view, Voronoi is one of the most similar designs for bone at present. Whether the Voronoi structure is one of the best designs can only be known through mechanical and biological experiments. The mechanical properties of the Voronoi scaffold were simulated by calculation. Moreover, the satisfied Young’s modulus and compressive strength were obtained in the results (Gómez et al., 2016). The Voronoi model was needed to be printed out through AM and compressed for its mechanical properties in further research. The compression test of the Voronoi scaffold was explored in the research of Maliaris and Sarafis (2016). In their study, the critical load of the Voronoi designed porous structures was increased by nearly 300%, which showed the ability for internal load-bearing implant structure. For another model built with algorithm, the results also showed that the manufactured model exhibited a high load to weight ratio, which is a crucial parameter for the scaffold (shown in Figure 2D) (Sharma et al., 2019). Voronoi designed scaffold with gradient porosity (60% to 95%) also showed excellent mechanical properties. A similar conclusion was developed in porosity ranging from 50% to 85%. In these compression test results, Young’s modulus and compressive strength were performed similarly to that of cancellous bone (Du et al., 2020). The mechanical properties of the Voronoi scaffold were studied by the FEA of different density gradient scaffolds. The results of the FEA illustrated that the stress of the gradient Voronoi scaffold was small at high porosity and large at low porosity. It indicated that the gradient Voronoi scaffold could be designed similar to natural bone in biomechanical properties (Wei et al., 2019). The mechanical properties of Voronoi scaffold were shown in Table 1.

These studies showed that the Voronoi designed scaffold in mechanical properties might not be less than the geometry-based scaffolds. As opposed to conventional geometry-based porous structures, the Voronoi scaffold had remarkable fluid properties. The computed fluid properties of Voronoi models directly were depended on being total porosity and bone surface area (Gómez et al., 2016). Thanks to the favorable fluid properties, Voronoi design could be used to excellent cell adhesion, migration, and, ultimately, bone ingrowth. In 2019, Liang et al. (2019) focused on the *in vitro* experimental of the Voronoi scaffold. The results identified that the excellent bone ingrowth property in the Voronoi scaffold. Their research indicated that the positive correlation was between the degree of pore irregularity and bone ingrowth (Liang et al., 2019). However, there were still few *in vivo* experiments that had been done in current researches. The *in vitro* and *in vivo* behavior of the 3D Voronoi scaffold remained to be further studied.

The Voronoi design has resembled cancellous bone in terms of bionics, mechanical properties, and bone ingrowth. It still had the common fault of cross structure, that is, the stress change of Voronoi was concentrated on the intersection of structs (Maliaris and Sarafis, 2016). Furthermore, in manufacturing, applying Voronoi design to orthopedics was an aporia. On balance, the Voronoi was most similar to the cancellous bone in morphology and mechanics.

#### TPMS

The majority of the existing designs were based on geometry with straight edges and sharp turns from Boolean intersections of geometric primitives. Suitable biomorphic environments for cell attachment, migration, and proliferation were not provided in these sharp areas (Nazir et al., 2019). Structures with TPMS, namely skeletal-TPMS and sheet-TPMS, consist of repeating elements with the minimum possible area. TPMS structures are smooth infinite surfaces, in which space is divided into two disjoint sub-volumes in free of self-intersections. Moreover, they are periodic in three independent surfaces (the concave and convex curvatures are symmetrical at all points) (Feng et al., 2019). The biomorphic geometry that was best imitate the secundum-natural substrate would be continuous through space surface and be divided into two not-necessarily equal sub-spaces by a non-intersecting two-sided surface. The structure of the TPMS is consistent with this argument. TPMS is an excellent structure for cell proliferation in theory (Afshar et al., 2016).

Research on skeleton TPMS of the orthopedic scaffold was extremely few. In 2019, a study on the selection of skeleton-TPMS was deserved attention. In research, the manufacturability, mechanical properties, and bone ingrowth of four kinds of skeleton-TPMS were compared. The results illustrated that Gyroid skeletal-TPMS had the most flexible design space, that is, it performed well in three aspects (Barba et al., 2019). However, the compressive strength of sheet-TPMS was 1.3–2 times that of skeleton TPMS, and its toughness was also higher (Cai et al., 2019).

There are commonly used types of sheet-TPMS as a list, which shown in Figure 1I. They can be, not limited to, primitive surface (P surface), Diamond surface (D surface), Gyroid surface (G surface), and I-WP surface. TPMS had a strict mathematical equation, which may be used to vary parameters of the structure to control the properties of the structures (Abueidda et al., 2017; Yuan et al., 2019).

Gyroid:

c⁢o⁢s⁢(x)⋅s⁢i⁢n⁢(y)+c⁢o⁢s⁢(y)⋅s⁢i⁢n⁢(z)+c⁢o⁢s⁢(z)⋅s⁢i⁢n⁢(x)=t

Diamond:

sin(x)⋅sin(y)⋅sin(z)+sin(x)⋅cos(y)⋅cos(z)+cos(E)⋅sin⁡(y)⋅cos⁡(z)+cos⁡(x)⋅cos⁡(y)⋅sin⁡(z)=t

P surface:

c⁢o⁢s⁢x+c⁢o⁢s⁢y+c⁢o⁢s⁢z=t

I-WP surface:

2⁢(c⁢o⁢s⁢x⁢c⁢o⁢s⁢y+c⁢o⁢s⁢y⁢c⁢o⁢s⁢z+c⁢o⁢s⁢z⁢c⁢o⁢s⁢x)-(c⁢o⁢s⁢ 2⁢x+c⁢o⁢s⁢ 2⁢y+c⁢o⁢s⁢ 2⁢z)=t

According to the deformation mechanism of TPMS, it can be divided into two main categories. One is belonging to the surface dominated by stretchings, such as the I-WP surface and P surface. Another is the domination of bending deformation, such as D surface and G surface (Afshar et al., 2016, 2018; Montazerian et al., 2017). The categories were shown in Figures 1I,J.

These four kinds of TPMS had mechanical properties matching with cortical and cancellous bone. Through the compression test of these four TPMS, it was shown that they exhibited a unique combination of relatively low Young’s modulus and high yield stress, which could avoid stress shielding (Bobbert et al., 2017). The porous scaffolds with controllable pore distribution could be built by the TPMS modeling method. The results of the FEA showed that the mechanical properties of TPMS simulation scaffolds were close to the bone’s expectation (Shi et al., 2018). At high porosity, these four structures all had Young’s modulus similar to that of cancellous bone (Yang et al., 2019a). Moreover, what is amazing was that the scaffold designed with P, I-WP has higher compressive performance than cancellous bone (Montazerian et al., 2017; Maskery et al., 2018a). At low porosity, the scaffold designed with P and D surface had Young’s modulus similar to that of cortical bone. Yan et al. (2015) evaluated Young’s modulus of P and D surfaces with low porosity. The results showed that the P and D surfaces with 5–10% porosity had the same Young’s modulus as cortical bone (Yan et al., 2015). A study on the effect of tuning the parameter (*t*) used in the gyroid equation, on the scaffold porosity and specific surface area, was carried out. With the increase of *t* (≤1.41), the porosity and specific surface area were increased. In mechanical tests, the modified G surface with 80% porosity (*t* = 1.41) had a similar compressive strength to cortical bone (Tripathi et al., 2019).

Castro et al. (2019b) assessed the mechanical properties of G surfaces with two porosity (50 and 70%). The scaffold stiffness function of porosity was determined by the asymptotic homogenization method and confirmed by mechanical testing. The results indicated that the G surface was a suitable design for bone tissue engineering (Castro et al., 2019b). The scaffold designed with these two classifications of TPMS was both suitable for orthopedic implants in the mechanic view, but the differences between the two classifications of TPMS needed to be discussed. The mechanical properties of TPMS family scaffolds were shown in Table 1.

Some differences were in these two deformation types of TPMS. Afshar et al. (2016) tested the mechanical properties between the stretching structure represented by P-surface and bending structure represented by D-surface. The results indicated that the stretching structure performed better in mechanics (Afshar et al., 2016). In the FEA, the same conclusion can be obtained. The Young’s modulus of stretching structure was twice the bending structure in the FEA results (Maskery et al., 2018b). The analysis of the failure mechanism can understand the difference in mechanical performance between two classifications of TPMS. The failure mechanism of the stretching structure represented by the P and I-WP surface was discussed in the FEA. The results showed that the P and I-WP surfaces were destroyed layer by layer under ultimate pressure (Abueidda et al., 2017; Afshar et al., 2018). The reason for its results was demonstrated in the research of Maskery et al. (2018b). The results showed that the stress concentration point of the P surface was the thin neck regions between the cell regions (Maskery et al., 2018b). The failure mechanism of the bending structure showed that the scaffold presented a 45° shear band under ultimate pressure (Afshar et al., 2016; Montazerian et al., 2017, 2019; Yang et al., 2019b). The reason for this result might be related to the uniform stress distribution on the D surface (Maskery et al., 2018a). Thus, shearing of the linkages was the predominant deformation mode in bending structure, while the axial deformation was seen in stretching structures. These results showed that the stretching structures had a high load-bearing capacity under uniaxial pressure.

The contrast between TPMS and other ways manufactured structure is well worth studying. There was an interesting finding that the actual porosity of TPMS was consistent with the design. The porosity of the cube was lost a part in the process of production. In Zaharin et al.’s (2018) research, the G surface structure was higher than the cube in mechanical properties in a compression test. Zhang L. et al. (2018) supported the conclusion. In the research of Zhang L. et al. (2018), the comparison of mechanical properties was found in the compression test. The results showed that the TPMS sheet structures are found to exhibit superior compressive strength compared to BCC cells (Zhang L. et al., 2018). Von Mises stress distributions in FE compression simulations of G, D, and P surfaces were shown in Figure 2E. Al-Ketan et al. (2018) discussed the mechanical properties between TPMS and strut-based structure in the same way. Their study indicated that the TPMS structure showed excellent mechanical properties in all tested structures. The research of Du Plessis et al. (2018) was different from the above conclusion. The strut-based models included Rhombic dodecahedron, Diamond, Gstruct, and Octet. The TPMS designs were P surface, G surface, I-W surface, and P surface. There were no significant differences in any of the mechanical properties between strut-based and TPMS designs (Du Plessis et al., 2018). In 2019, a significant study was completed by Guo et al. (2019). They compared TPMS with its similar struct-related structure. The similar Young’s modulus and compressive strength ware in TPMS structure and similar struct structure, and TPMS structure exhibited more uniform and smooth transition stress distribution (Guo et al., 2019). In general, the mechanical properties of the TPMS structure were no less than that of the strut-based structure.

There were few studies *in vivo* and *in vitro* of TPMS structure. The specific surface area and permeability can be used to predict bone ingrowth. The specific surface area was an important parameter to determine the cell adsorption area. It could be seen in the results that the D surface had the highest specific surface (Guo et al., 2019). At the same time, many pieces of research indicated that the G surface had the highest permeability, which means adequate oxygen and nutrition delivery (Montazerian et al., 2017, 2019; Yang et al., 2018; Castro et al., 2019a; Ali et al., 2020). Therefore, the D and G surface might have the best bone growth, which needs to be verified by future biological experiments. In 2019, a preliminary cell migration experiment was as confirming this view, adipose-derived mesenchymal stromal cells migrated furthest on scaffolds with Diamond, followed by Gyroid. The D and G surfaces are still rare on *in vitro* and *in vivo* researches (Valainis et al., 2019). The reason for less bone ingrowth experiments on D or G surface might be that compared with other TPMS, the manufacturing accuracy of the D surface was relatively low. P surface had ample design space and high manufacturing accuracy. The effect of porous Ti on bone ingrowth was studied by using the P surface designed scaffold. Through the push-out test, it showed that the scaffold has an excellent bone ingrowth effect (Shi et al., 2019).

In general, the TPMS is an excellent choice for orthopedic scaffolds. The four types of TPMS are close to the data of natural bone in terms of porosity rate, Young’s modulus, compressive performance, and permeability. Stretching structure has excellent mechanical properties, while the bending structure has good performance in permeability. A new TPMS based scaffold could be proposed, that is, the outer layer is stretching the TPMS structure, and the inner layer is bending the TPMS structure.

#### Other Fields

Excellent mechanical properties and permeability are necessary for orthopedic scaffolds. In nature, the high compressive strength and low weight are in many structures, which is essential for orthopedic implants. With the natural structure as reference, it might be expected to design and manufacture orthopedic scaffolds with excellent mechanical properties. Random structure means lower Young’s modulus and better permeability than a regular structure for orthopedic implants. Random design in other fields might be used in orthopedics.

**FIGURE 3:**
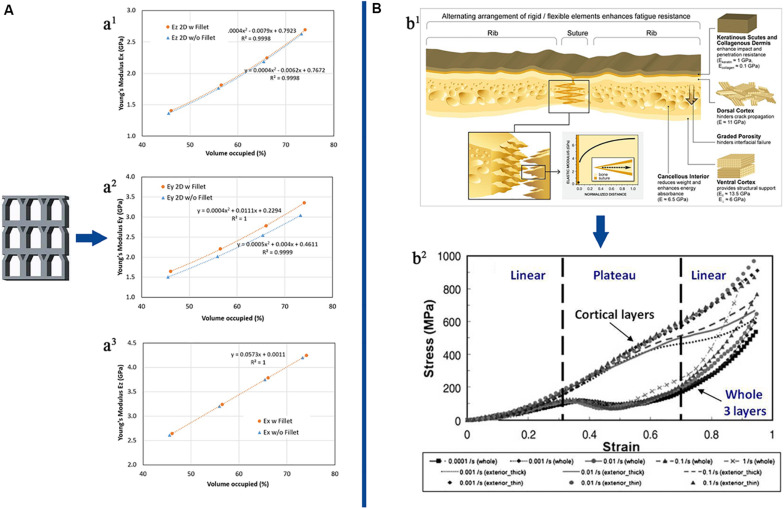
The other field designs and their mechanical property. **(A)** 2D cuttlefish bone model and its mechanical property. Young’s modulus for cuttlefish bone 2D model: **(a^1^)** Ex (planar), **(a^2^)** Ey (planar), and **(a^3^)** Ez (thickness). **(B)** The turtle carapace structure and its mechanical property. **(b^1^)** The various microscopic features of the turtle carapace, including the layered rib structure, the perisuture, and keratin scutes. The elastic moduli shown were calculated from nanoindentation measurements performed under wet conditions, reflecting physiological conditions. **(b^2^)** Representative quasi-static compressive performance of dry ribs taken from carapaces of the box turtle (*Terrapene carolina*). The specimens, containing the whole three layers, or alternatively only individual cortex layer, were tested under various strain-rates. Specimens containing the whole three layers show a unique deformation behavior involving a pronounced plateau region, corresponding to buckling and fracturing of the trabeculae forming the cancellous interior. Reprinted: **(A)** from Hu et al. (2017) with permission from Elsevier; **(B)** from Achrai and Wagner (2017) with permission from Elsevier.

The turtle shell structure, in nature, is designed to protect from predator predation. It may be superior to traditional impact-resistant materials and may bring new mechanical insights. (The turtle shell structure was showed in Figure 3B). Achrai and Wagner (2017) reviewed the microscopic features of the turtle carapace structure and how to control its various macroscopic mechanical responses related to protection functions, including dynamic (impact and cyclic) compression and bending loading. The results showed that the tortoiseshell structure had superior compression and bending resistance. As orthopedic scaffolds, the graded porosity was essential. The turtle carapace also exhibited functionally graded structural elements, such as graded porosity and graded mineralization. The tortoiseshell structure might be a direction of orthopedic development in the future (Achrai and Wagner, 2017).

Random cell distribution was one of the factors that affected the growth performance of scaffold bone (Liang et al., 2019). A Japanese study has shown that a random porous structure was generated based on Monte Carlo simulation. A set of hierarchical point clouds was created, which allows the solid model generated by the point cloud to present a randomly distributed porous structure in that study. In this study, the resulting structure is relatively simple, a ring-shaped random porous structure, but according to theory, complex structures were being explored. It is worth exploring from the perspective of the orthopedic surgeon for this randomly distributed porous structure (Sharif Ullah, 2017).

Hu et al. (2017) conceived a future cellular structure, that is, the 2D cuttlefish model. In their study, the 2D cuttlefish model had an excellent compressive capacity in the vertical direction, which may be related to the high pressure in the deep sea. The cuttlefish model structure was shown in Figure 3A. Using the additive manufacturing technology makes the 2D cuttlefish model into a 3D structure; this will provide a new idea for future porous materials, orthopedics maybe its beneficiaries. The cuttlefish model had high-pressure resistance ability while its weight was light. This kind of high load ratio was indispensable for the orthopedic scaffold, so it might be a feasible choice to apply the cuttlefish model to porous structure (Hu et al., 2017).

There are various other designs in nature or other fields that are meaningful for orthopedic implants. Here is just an idea to focus on other areas rather than just imitating the structure of bone.

## The Whole Design

The cellular designs were detailed discussed in the other part of the text. The whole design is also well worth exploring. The whole design cannot be ignored as well for the entire structure in mechanical property and biological aspects. The macroscopic model is divided into four categories according to the requirements of orthopedic scaffolds, uniform design, layered gradient design, continuous gradient design, and design based on TO.

### The Uniform Design

**FIGURE 4:**
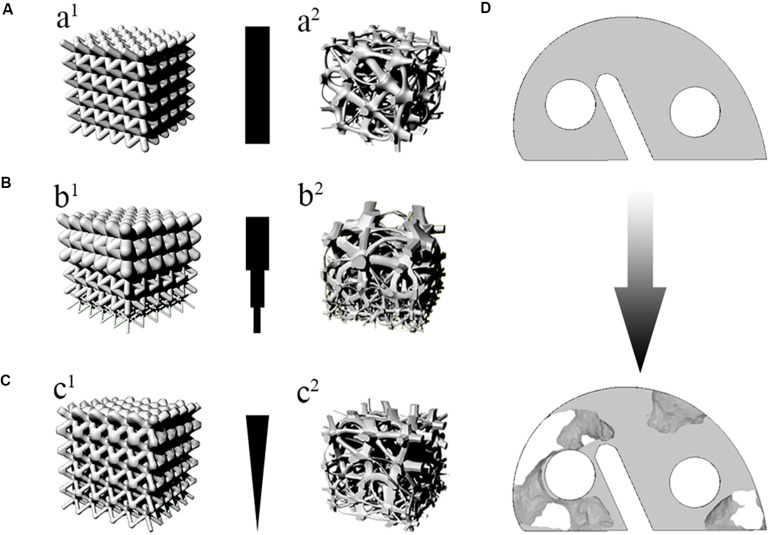
The classification of whole design. **(A)** The uniform design. **(a^1^)** e.g., BCC. **(a^2^)** e.g., Voronoi. **(B)** The layered Gradient design. **(b^1^)** e.g., BCC. **(b^2^)** e.g., Voronoi. **(C)** The continuous gradient design. **(c^1^)** e.g., BCC. **(c^2^)** e.g., Voronoi. **(D)** The design based on TO. Taking the knee joint gasket as an example to describe the design based on TO.

Uniform structure design means that the porosity of the whole scaffold is the same. The schematic is shown in Figure 4A. In the early stage, due to the limitation of printing accuracy and bone structure research, the uniform structure became the only choice. In the early stage, various complex structures were difficult to be manufactured. Smooth surfaces were manufactured with AM technology, which was not possible in the early stage. The TPMS structure had just been effectively generated in 2011 (Hao et al., 2011).

Porosity and cell unit types were essential factors affecting the mechanical properties of scaffolds. The type of unit cell was detailed in the above. Scaffolds that were suitable for orthopedic implants should have the same porosity and mechanical properties as human bone. In order to simulate the structure of bone, the porosity of uniform scaffolds was designed in two different situations. One situation was a high porosity scaffold simulating cancellous bone. Another approach was developed for simulating cortical bone to adopt the low porosity scaffold. Wieding et al. (2015) had manufactured a porous Ti6Al4V scaffold with 60–80% porosity, and its elastic modulus was 6–8 GPa. Both porosity and Young’s modulus were remarkably similar to cancellous bone (Wieding et al., 2015). Li et al. (2016) studied the relationship between porosity and mechanical properties of scaffolds. It was feasible to reduce Young’s modulus to the range of cancellous bone while increasing porosity (Li et al., 2016). Similarly, a model of varying Young’s modulus by adjusting porosity was established by Kadkhodapour et al. (2015). Their study suggested that Young’s modulus similar to bone could be obtained by adjusting the porosity from 65% to 90% (Kadkhodapour et al., 2015). In addition to changing the porosity to change the mechanical properties of scaffold, it can also increase the overall compressive capacity by adding reinforcement outside the scaffold (Xia et al., 2019).

The mechanical properties of porous structure and bone were compared in the research of Yan et al. (2015). In their study, Young’s modulus of porous TPMS resembled cortical bone (5–10%), and its compressive strength was much higher than cortical bone (Yan et al., 2015). Torres-Sanchez et al. (2017) explored similar research, in which two different types of porous scaffolds were designed. One was to imitate cortical bone with low porosity. The other type was to imitate cancellous bone with high porosity. In their research results, the optimal scaffold for cortical bone implanting has a porosity value of 27–37%; the optimal scaffold for trabecular bone implanting had a porosity value of 54–58% (Torres-Sanchez et al., 2017). Research about Young’s modulus of porous Ti6Al4V was developed through FEA. The mechanical properties of different porosity were measured in the research. The results showed that Young’s modulus of 19.59–1.85 GPa could be obtained by adjusting the porosity from 50 to 80% (Golodnov et al., 2018). It was practicable to change the mechanical properties of the porous structure by adjusting the porosity.

The porosity affects not only the mechanical property but also the bone ingrowth (Yan et al., 2015; Hedayati et al., 2016b). The previous research suggested that over 60% porosity and pore size larger than 300 μm could promote bone formation (Hollister et al., 2005; Roosa et al., 2010; Li et al., 2016). The *in vitro* research of Li et al. (2018b) was supported this opinion. Their study illustrated that the scaffolds with 65% porosity showed a significant bone ingrowth property (Li et al., 2018b). Torres-Sanchez et al. (2017) evaluated the effect of porosity on bone ingrowth. In their results, due to the suitable pore size (551 ± 55 μm) and porosity (74 ± 4%), the structure was expected to be suitable for bone ingrowth (Torres-Sanchez et al., 2017). The effects of different porosity (60% and 70%) and pore size (500 μm, 600 μm, and 700 μm) on bone formation and cell proliferation were compared. The results showed the scaffold with a pore size of 500 μm and porosity of 60% showed the best cell proliferation and osteogenic differentiation (*in vitro* experiments) and bone ingrowth (*in vivo* experiments) (Chen et al., 2020). Nune et al. (2017b) discussed biological activity and osteoblast function in porous scaffolds. Their study showed the scaffold with 70% porosity provided a pathway for communication and maturation to differentiated phenotypes between cells (Nune et al., 2017b). These studies suggested that the porosity of suitable bone was more than 60%. The scaffold with high porosity has good bone ingrowth effect, and the internal reason might be its high permeability (Mitsak et al., 2011; Dias et al., 2012).

However, living tissues are the non-homogenous structure, which is composed of different biological and functional layers, coexisting in hierarchy and harmony. Reconstruction of heterogeneous tissue with a homogeneous scaffold may lead to suboptimal results.

### Layered Gradient Design

Owing to the layered structure of bone (Lerebours et al., 2015), the idea of designing scaffold as the layered gradient was put forward. Layered gradient design is to design the scaffold with low to high porosity in different layers to imitate the cortical bone to cancellous bone. The layered gradient design is shown in Figure 4B, which used BCC and Voronoi as example. The difference between uniform and gradient structures has been explored.

The layered gradient structure can be designed in various methods. Shi et al. (2017) made a three-layer gradient scaffold through changing struct diameter, in which the porosity was from 68.5% to 88.2% with the Young modulus of 12–18 GPa. Another method was to connect the completely dense layer and porous layer to form a gradient scaffold (Di Luca et al., 2016; Fousova et al., 2017). By changing the diameter of the rhombus cell struts, the scaffold was made into three different porosity and pore (Zhang et al., 2019). The morphological parameters, permeability, and mechanical properties of layered gradient scaffolds were measured and compared with porous biomaterials based on the same cell type. The results showed that the mechanical properties of the scaffold were better than the scaffold with a uniform porous structure. These researches suggested that the mechanical properties were suitable for bone implants in layered gradient scaffold.

With the change of pore distribution, Young’s modulus of scaffold changed in gradient. The *in vitro* experiment also confirmed that the gradient structure is more conducive to the biological fixation of scaffolds. The gradient structure was manufactured to imitate the bone structure used three layers of different porosity. The outer layer had a porosity of 29.6% ± 5%, and the middle and inner layers had a porosity of 50.8% ± 8.1% and 77.6% ± 3.2%, respectively, which resembled the gradient in the bone. The human mesenchymal stem cells (hMSCs) were accepted in order to evaluate the bone ingrowth *in vitro*. The hMSCs behavior was analyzed in terms of growth, extracellular matrix deposition, and differentiation toward the osteogenic lineage. The results showed that alkaline phosphatase (ALP) activity was higher in the high porosity of the same scaffold. Their study suggested that porosity can influence the differentiation of hMSCs into osteoblasts (Di Luca et al., 2016). Another study investigated the behavior of hMSCs in different porosity regions of gradient scaffolds. The results illustrated that ALP activity and calcium content were increased with increasing porosity. Their research suggested that the gradient porosity scaffold was an appealing structure to support gradual osteogenic differentiation of adult stem cells (Nune et al., 2017a). An exceptional design in the layer gradient could be expected that the regular cube in the core to increase the mechanical property, the irregular design in outer to promote the bone ingrowth (Dallago et al., 2018). The Pivonka group applied the mathematical multiscale model in the research of bone reconstruction (Pivonka and Dunstan, 2012; Scheiner et al., 2013). In 2018, they proposed a mathematical model, which can simulate the evolution of bone tissue under various loading conditions well. They indicated that the biological cells and biological factors driving bone remoting were actually located in differently sized pore spaces (Pastrama et al., 2018). It is suggested that the layered design in future research might consider the different cells located layers to design.

These studies showed that layered gradient design was superior to uniform structure in both mechanical and biological properties. However, none of these researches showed the theoretical basis of stratifying.

### Continuous Gradient Design

In most cases, the layered structure is the best structure to simulate bone. However, the layered gradient structure, the connection between layers, and the stress transition between layers are all to be solved. The idea of continuous gradients came into being. Now part of the continuous gradient design is still in the design stage.

**FIGURE 5:**
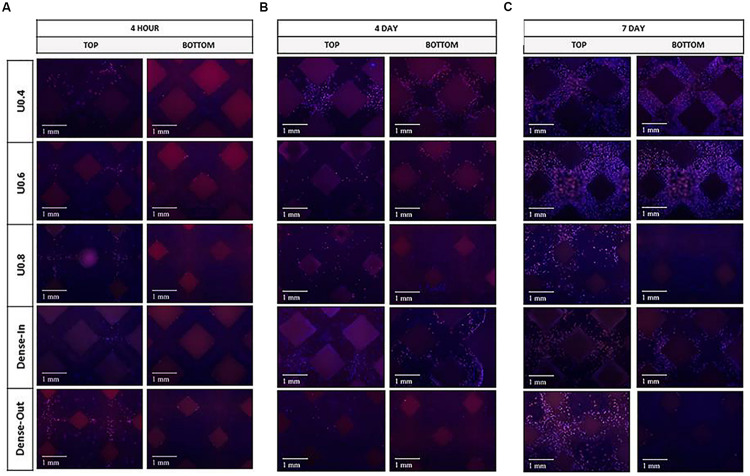
Fluorescence micrographs on the unit and gradient BCC. Fluorescence micrographs representing merged Hoechst stained nucleus (blue) and actin cytoskeleton (red) of preosteoblast cells on the uniform and gradient BCC structures after culturing for **(A)** 4 h, **(B)** 4 days and **(C)** 7 days. The top represents the side where cells were seeded onto the samples. Reprinted from Onal et al. (2018) under the terms of the Creative Commons Attribution License.

**FIGURE 6:**
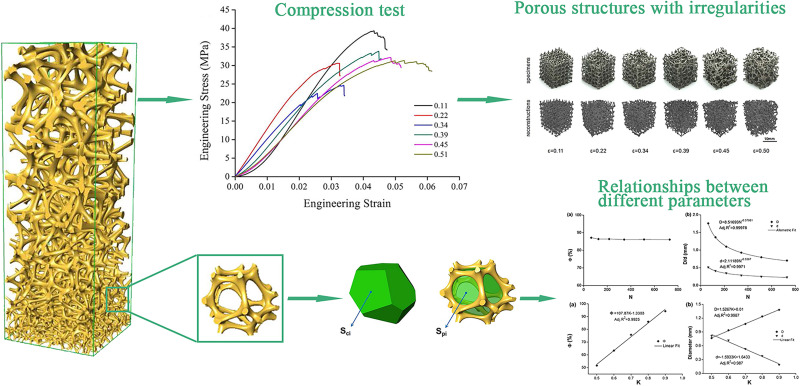
The relationships of various parameters in gradient Voronoi structure. Schematic showing the unit cell of gradient Voronoi scaffolds, the relationships between parameters, the mechanical properties in different porosity, and the porous structures with irregularities. Reprinted from Wang et al. (2018) with permission from American Chemical Society.

There are several methods to get the effect of the continuous gradient. One of the continuous gradient structures were generated by gradually changing the strut diameter of a BCC unit cell (Onal et al., 2018). The schematic diagram of this structure was shown in Figure 4c^1^. In this research, the results indicated that optimal gradient structures should possess small pores in the core to increase mechanical strength while large pores should be utilized in the outer surface to enhance cell penetration and proliferation. The continuous gradient structure had excellent osteogenic performance. The underlying reason was that it provided a way for the effective transfer of nutrients from the large pore side structure to the other end (small pores), leading to the generation of mineralized extracellular matrix by differentiating pre-osteoblasts. The growth rate of osteoblasts brought by this gradient structure is higher than that of uniform structure (shown in Figure 5). With AM rapid development, some individual unit cells on the algorithm are constituted to the continuous gradient structure. As we can see, the continuous gradient Voronoi scaffolds were reported. An innovative method based on the probability sphere was used in the article, which was controllable based on the probability sphere. The diagram of the continuous gradient Voronoi is shown in Figure 4c^2^. Through the quasi-static compressive test, the results showed that continuous scaffolds had approving porosities ranging from 60 to 95% and pore size ranging from 200 to 1200 μm, which conformed to human bone microstructure. The compressive properties of gradient Voronoi structures were able to be adjusted to the level of cortical bone that the young modulus varied from 0.14 to 2.37 GPa, and the compressive strengths varied from 1.94 to 116.61 MPa (Wang et al., 2018; Du et al., 2020). The relationships of various parameters in gradient Voronoi structure were showed in Figure 6. The TPMS was also developed rapidly because of the improving accuracy of AM. Li D. et al. (2019) reported a design method for TPMS-based cellular structure. The FEA of the designed and optimized structure reveals its excellent mechanical properties (Li D. et al., 2019). In the research of Ma et al. (2020), the gradient TPMS scaffolds were generated by adjusting the parameter t in the TPMS equation to change porosity. In the compressive test results, compared to uniform TPMS scaffolds, the gradient TPMS scaffolds exhibited improvements in elasticity, strengths, and especially energy absorption (Ma et al., 2020). A continuous gradient G surface TPMS with varying gradient directions indicated by Yang et al. (2019a). The results showed that the Young modulus (898.05–1165.49 MPa) and compressive strength (15.82–27.83 MPa) which were similar to cancellous bone, expressed in this gradient structure (Yang et al., 2019a).

The mechanical properties of the continuous gradient scaffolds were changed continuously with the gradient, and Young’s modulus was also within the range of bone, while the compressive strength was higher than the bone. Although there was a paper indicated that the bone ingrowth was performed excellently. The bone ingrowth or osteoblast proliferation was still studied scanty in the continuous gradient scaffolds.

### Design Based on TO

Topological optimization is defined that optimizing the material distribution in the design field, and the material layout is optimized on the fixed finite element mesh. TO can be divided into two types. One is the discrete approach. Another is the continuum approach. In the medical field, it is rare to use a discrete approach. The continuum method is a micromechanics theory-based approach that considers the design space as an artificial composite material with a large number of periodically distributed small holes. In the finally optimized model, small hole regions are filled (solid), whereas areas with large apertures are considered empty (no material) (Ruben et al., 2012; Tang and Zhao, 2016; Savio et al., 2017).

In 2000, the Hollister group presented an innovative study at that time. An image-based approach was proposed to design and manufacture patient-specific craniofacial biomaterial scaffolds directly from CT or MRI data. The image-based approach to designing scaffolds provides for much faster creation of scaffold designs. In this approach, voxel density distribution was used to define scaffold topology (Hollister et al., 2000, 2002; Lin et al., 2004; Hollister, 2009). With the progress in computer technology, the TO was combined with FEA. The stiffness requirements of the scaffold in different areas were obtained as the results of the scaffold FEA on Macroscopic structure. The TO distributed more material (high density) in high-stress regions such that the stiffness of the system was maximized for a given material volume fraction (Kang et al., 2012, 2013; Al-Tamimi et al., 2017). Various mesh units can be assigned to different density regions. A numerical homogenization based TO was applied to the design of three-dimensional unit for tissue engineering scaffolds in the research of Kang et al. (2010). The cross-property bounds between bulk modulus and diffusion coefficient was used to determine the unit structure of a given porosity (Lin et al., 2004; Kang et al., 2010; Dias et al., 2014; Coelho et al., 2015; Knutsen et al., 2015). The designed high porosity units were used in the low-stress area, while the low porosity units were used in the high-stress area (Kang et al., 2010). TPMS can also be used as a TO basic unit in the research of Li D. et al. (2019). TPMS-based cell structure and an optimization algorithm to calculate the optimal relative density distribution of the target shown by their study. Through finite element analysis and experiments, the structure was more rigid than uniform cell structures (Li D. et al., 2019). A mesh topology optimization framework is proposed to minimize the total weight of the structure under stress constraints, that is, to form a gradient structure used G surface as basic TO unit. In the experimental results, the proposed optimization framework is sufficient for the design examples studied, which can significantly improve the mechanical properties of the structure (Li D. et al., 2018). Cheng et al. (2019) proposed a new method of designing a gradient structure through topology optimization under stress constraints in 2019. Panesar et al. (2018) indicated that the gradient structure based on TO strategy was identified as the most robust strategy from a mechanical property standpoint due to its high resilience to loading variabilities. On a macroscopic scale, the scaffold imitated complex bone internal structure was recovered using a perimeter control based TO approach in the proof of concept of Park et al. (2018). Nevertheless, it only dealt with simplified 2D problems, and the 3D model was still not to involve (Park et al., 2018). Taking the knee joint metal block as an example, the metal block after TO was shown in Figure 4D.

The topology optimization technology in orthopedics can make the stress distribution and porosity of scaffold more reasonable to avoid the stress shielding effect.

## Challenges and Future Directions

The application of porous structures in the design and manufacture of orthopedic scaffolds have a broad future. In order to increase the use of these structure in the orthopedic scaffold substantially, several challenges need to be addressed:

• More attempts are needed to be made in the cellular structure. The existing cellular structure could be defective, which is unable to simulate the structure of the bone completely. More random mesh structures need to be discovered and manufactured.
• The fatigue life of most porous structures is still uncertain in current research. Further tests on fatigue life should be carried out under the guidance of design.
• All the porous structures manufactured by AM should be put together for comparison to evaluate the different applications of different cellular structures in different orthopedic directions. Moreover, the properties of all microscopic units of the porous structure should be tested under the same conditions.
• The feasibility of a gradient structure needs to be considered. More expansion should be needed in the overall design, especially the design of the gradient structure requires more ideas. The different application scene of the continuous design and layered design in gradient design may become the research hotspot.
• The limitations of AM technology need to be considered. AM technology has some accuracy problems with the manufacture of complex porous structures. In design, the error of different printing technologies needs to be considered.
• The biological properties of the porous structure need to be evaluated. Compared with mechanics experiments, *in vitro* and *in vivo* experiments were performed less, especially long-term *in vivo* experiments are a scanty few.

## Conclusion

This paper reviews the different porous structure designs, including microscopic cellular structure design, the whole design, and other fields which may be used in the orthopedic design, and attempts to identify areas for research and future research directions. Besides, the biological properties, mechanical properties, and deficiencies of the porous are also described. The following is a summary of the findings. In the non-parametric designed scaffolds, most of the structures are similar; all of them are small changes to simple structures. The most existing microscopic cellular structures are designed to focus on cube-based structures while ignoring random structures and non-cube structures. Thus, the microstructure needs to be thoroughly summarized. At present, the development of TPMS and Voronoi scaffolds is increasing. The superior randomness is shown in Voronoi designed scaffolds, which is close to the performance of cancellous bone. Due to the continuous smooth surface, excellent permeability is expressed in the TPMS scaffolds. These two designs have elements that are necessary for the orthopedic stent. However, *in vivo* experimental validation and *in vitro* experiments are lacking for these new structures. In nature or other fields, various designs possess the necessary properties for orthopedics. Perhaps focusing on other structures is also a practical option, not just to simulate bone structures. In terms of whole designs, the gradient is better than the uniform. The future direction is the gradient structure, which is similar to the macrostructure of bone. Furthermore, the gradient is divided into many types. Generally speaking, the continuous gradient is better, but more research is needed to illustrate.

In summary, since the random structure in the microstructure is superior in biological properties, while ensuring mechanical properties, and the macrostructure gradient structure is considered to be a better choice. An idea was put forward by us in which gradient design is used in the whole design while the irregular microporous design is adopted in the microscopic design.

## Author Contributions

HC contributed to the conceptualization and writing – original draft. QH contributed to the writing – review and editing. CW contributed to the project administration and investigation. YL contributed to the investigation. BC contributed to the supervision and validation. JW contributed to the resources, supervision, and funding acquisition.

## Conflict of Interest

The authors declare that the research was conducted in the absence of any commercial or financial relationships that could be construed as a potential conflict of interest.
